# Supplementary material for: Observation of a transient intermediate in the ultrafast relaxation dynamics of the excess electron in strong-field-ionized liquid water
Source: Nat Commun. 2022 Nov 26;13:7300. doi: 10.1038/s41467-022-34981-4 (PMC9701198; doi:10.1038/s41467-022-34981-4)
Supplement: Supplementary file 1 — Supplementary Information [file 41467_2022_34981_MOESM1_ESM.pdf]

SUPPLEMENTARY INFORMATION

Observation of a transient intermediate in the ultrafast relaxation dynamics of the excess electron in strong-field-ionized liquid water

Pei Jiang Low,[1,†] Weibin Chu,[2,†] Zhaogang Nie,[1] Muhammad Shafiq Bin Mohd Yusof,[1] Oleg V. Prezhdo,[2,*] Zhi-Heng Loh[1,*]

[1] School of Chemistry, Chemical Engineering and Biotechnology, Nanyang Technological University, Singapore

[2] Department of Chemistry, University of Southern California, Los Angeles, California, USA

[†] These authors contributed equally.

[*] Corresponding authors: prezhdo@usc.edu (O.V.P.), zhiheng@ntu.edu.sg (Z.-H.L.)

Supplementary Note 1: Pump-power dependence measurements

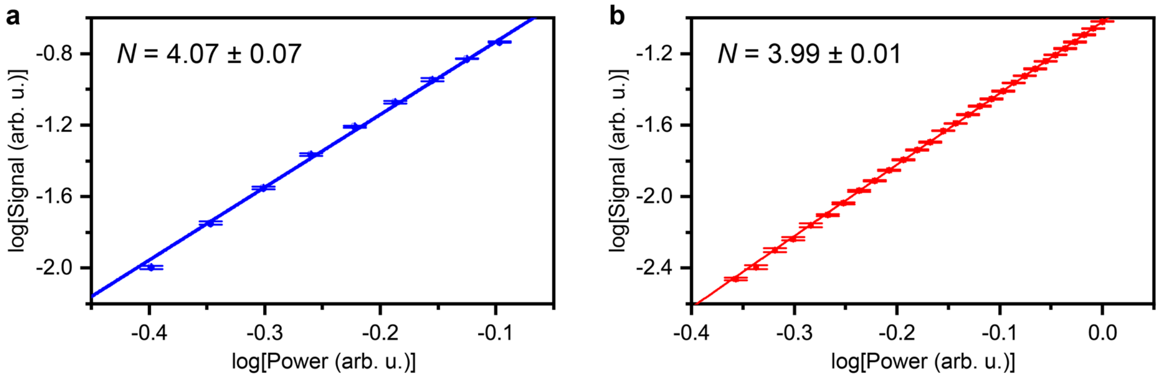

Supplementary Figure 1. Photon order measurement results. Pump power dependence recorded at probe wavelengths of **(a)** 700 nm and **(b)** 1.45 μm.

Power dependence measurements are performed to determine the photon order of the strong-field ionization process of liquid water. For an N-photon ionization process, the differential absorption signal, ΔA, scales with intensity, I, as $\Delta A \propto I^N$. The photon-order measurements are performed for the visible-NIR pump in both the broadband (528 – 919 nm) and narrowband (533 – 741 nm) configurations, probing the ΔA signal recorded at 1-ps time delay in the visible (700 nm, Supplementary Figure 1a) and SWIR (1.45 μm, Supplementary Figure 1b), respectively. The photon orders obtained with visible and SWIR probing are 4.07 ± 0.07 and 3.99 ± 0.01, respectively. Both values are consistent with a four-photon process, suggesting that ionization of water proceeds via the neutral electronically excited $\tilde{A}$ state, located ~8 eV above the neutral ground state[1].

Supplementary Note 2: Simulated time-resolved differential absorption spectra

Spectral simulations were performed to verify that the experimental differential absorption spectra support the existence of a short-lived intermediate. Simulated spectra with and without an intermediate state in the kinetic model are shown in Supplementary Figures 2a and 2b, respectively. The input parameters are obtained from Supplementary Table 1. It is evident that the appearance of a SWIR absorption at early time delays (Supplementary Figure 2a), on top of the continuous spectral blue shift, is a signature of the transient intermediate.

Spectral simulations were also performed to evaluate the effect of the experimental time resolution on the ability to observe the short-lived transient intermediate. Simulated spectra with a time resolution of 10 fs FWHM and 300 fs FWHM are shown in Supplementary Figures 3a and 3b, respectively. The input parameters are obtained from Supplementary Table 1. It is evident that a time resolution of 300 fs FWHM, similar to those employed in early studies, hampers the observation of the short-lived SWIR absorption signature, even in the absence of coherent artifacts.

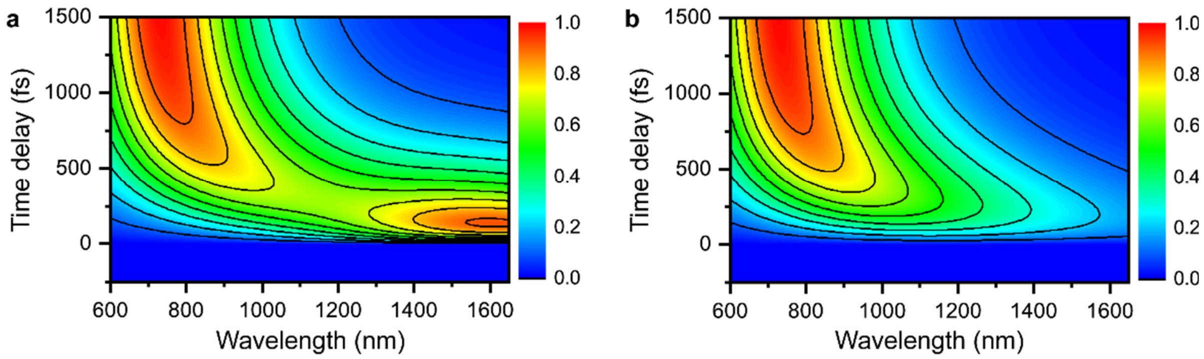

Supplementary Figure 2. Spectral signature of a transient intermediate. Comparison of the simulated time-resolved differential absorption spectra when the conduction-band electron relaxes **(a)** via the *p* state transient intermediate to the *s* state and **(b)** directly to the *s* state. The color scale is the differential absorption signal in arbitrary units. Note that both plots employ the same color scale.

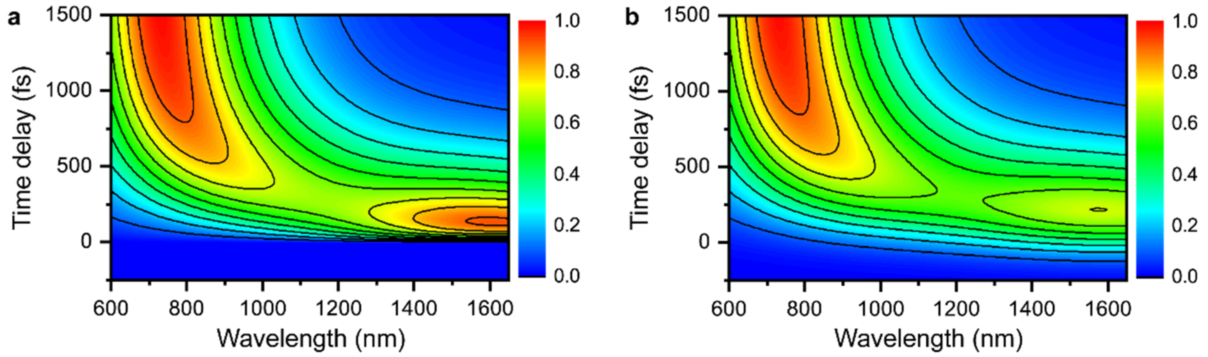

Supplementary Figure 3. Effect of experimental time resolution on the amplitude of the SWIR absorption. Comparison of the simulated time-resolved differential absorption spectra when the conduction-band electron relaxes via the p state to the s state, as observed with a Gaussian FWHM instrument response function of **(a)** 10 fs and **(b)** 300 fs. The color scale is the differential absorption signal in arbitrary units. Note that both plots employ the same color scale.

Supplementary Note 3: Theoretical model for the global fitting of experimental data

A visual representation of the model used to fit the data is shown in Fig. 1 of the main text. The mathematical formulation of the model that is used to fit the experimental data is adapted from ref. [2]. A few assumptions are made to avoid over-parameterizing the fit. First, the excess electrons in the conduction band state (e_{CB}) have a probability, P_{dir}, of transitioning directly to the vibrationally hot *s* state (e_s^*) and a probability, $1-P_{\mathrm{dir}}$, of transitioning to the p state (e_p). Second, the initial e_s^* state is the same regardless of the precursor state. Third, the absorption linewidths of the hydrated e_s electron and its vibrationally hot e_s^* state are assumed to be identical. Fourth, we ignore the solvation of e_p because the time-resolved differential absorption spectrum probed in the SWIR does not reveal any spectral shift that is indicative of solvation dynamics.

From sequential kinetics, the time-evolution of the e_s population is given by

$$N_s(t) = N_{CB}(0)\left[1 - P_{\mathrm{dir}}e^{-k_{\mathrm{CB}}t} + \frac{1-P_{\mathrm{dir}}}{k_p - k_{\mathrm{CB}}}\left(k_{\mathrm{CB}}e^{-k_p t} - k_p e^{-k_{\mathrm{CB}}t}\right)\right], \qquad \text{(S1)}$$

whereas that of the e_p population is given by

$$N_p(t) = \frac{(1-P_{\mathrm{dir}})N_c(0)k_{\mathrm{CB}}}{k_p - k_{\mathrm{CB}}}\left(e^{-k_{\mathrm{CB}}t} - e^{-k_p t}\right). \qquad \text{(S2)}$$

In the above equations, $N_s(t)$ and $N_p(t)$ correspond to the time-dependent populations of e_s and e_p, respectively, $N_c(0)$ is the initial population of excess electrons injected into the conduction band of liquid water by ionization, the e_{CB} population decays with rate constant $k_{\mathrm{CB}} = 1/\tau_{\mathrm{CB}}$ and the e_p population decays with rate constant $k_p = 1/\tau_p$, where τ_{CB} and τ_p are the corresponding lifetimes of e_{CB} and e_p, respectively. Since the maximum time delay that is employed in the global fitting is 1.5 ps, we do not consider in our analysis the depopulation of e_s via geminate recombination, a process that occurs on ~100-ps timescales under our experimental conditions[3-5].

The absorption lineshape of the hydrated e_s electron can be described by a Gaussian-Lorentzian lineshape[6]. Following ref. [7], we model the time-evolution of the absorption spectrum, reflecting the solvation of the e_s^* electron, by assuming that the center frequency shifts with time. Hence, the time-dependent absorption spectrum of the hydrated e_s^* electron takes on the following form.

$$\epsilon_s(E,t) = \begin{cases} \epsilon_{\max,s} \exp\left\{-\dfrac{\ln 2\,[E - E_s(t)]^2}{W_{G,s}^2}\right\}, & E \le E_s(t) \\ \dfrac{\epsilon_{\max,s}}{\left[\dfrac{E - E_s(t)}{W_{L,s}}\right]^2 + 1}, & E > E_s(t) \end{cases} \tag{S3}$$

In the above expression, $\epsilon_{\max,s}$ is the absorption coefficient of e_s at the peak, $E_s(t)$ is the center transition energy, and $W_{G,s}$ and $W_{L,s}$ are respectively the widths of the Gaussian and Lorentzian components. To avoid over-parameterizing the fit, $W_{G,s}$ and $W_{L,s}$ are assumed to be remain constant as e_s^* undergoes solvation. In our analysis, we employ the literature values $\epsilon_{\max,s} =$ 19700 M^{-1} cm^{-1}, $W_{G,s} = 0.843$ eV and $W_{L,s} = 0.488$ eV for H_2O, and $W_{G,s} = 0.802$ eV and $W_{L,s} =$ 0.453 eV for D_2O[6].

The absorption spectral shift of e_s^* as it undergoes solvation is modeled by the relation

$$E_s(t) = E_s(\infty) + [E_s(0) - E_s(\infty)]e^{-k_{\text{solv}}t}, \tag{S4}$$

where $E_s(0)$ and $E_s(\infty)$ present the initial and equilibrium peak absorption energies, respectively, and $k_{\text{solv}} = 1/\tau_{\text{solv}}$ is the rate of solvation and vibrational relaxation, characterized by the time constant τ_{solv}. The spectral evolution associated with e_s formation was previously modeled as a product of $E_s(t)$ and $N_s(t)$[7]. To correctly account for the solvation dynamics, however, it is important to realize that e_s^* produced from e_p at different time delays would have undergone different degrees of solvation. As such, the spectral evolution that is associated with the formation

of e_s^* and its subsequent solvation to yield e_s should be represented as a convolution of the population $N_s(t)$ with the time-dependent absorption lineshape $\epsilon_s(E,t)$, i.e.,

$$\Delta A_s(E,t) = \int_0^t \epsilon_s(E,t') \frac{dN_s(t-t')}{dt'} dt'. \tag{S5}$$

The above expression accounts for the birth of e_s^* at each time t' and its subsequent solvation, starting from t', to form e_s. Integrating each piecewise increase in e_s^* over t' up to time delay t yields the total response $\Delta A_s(E,t)$.

Following ref. [2], the absorption lineshape of e_p is similarly described by a Gaussian-Lorentzian function.

$$\epsilon_p(E) = \begin{cases} \epsilon_{\max,p} \exp\left[-\frac{\ln 2 \left(E-E_p\right)^2}{W_{\mathrm{G},p}^2}\right], & E \le E_p \\ \frac{\epsilon_{\max,p}}{\left(\frac{E-E_p}{W_{\mathrm{L},p}}\right)^2 + 1}, & E > E_p \end{cases} \tag{S6}$$

In the above, $\epsilon_{\max,p}$ is the absorption coefficient of e_p at the peak, $W_{\mathrm{G},s}$ and $W_{\mathrm{L},s}$ are respectively the widths of the Gaussian and Lorentzian components of the p state absorption lineshape. Since no discernible spectral shift is observed in the SWIR, we assume that the absorption lineshape does not evolve with time. The absorption signal that originates from e_p is thus

$$\Delta A_p(E,t) = \epsilon_p(E) N_p(t). \tag{S7}$$

Finally, the total absorption signal is

$$\Delta A_{\mathrm{total}}(E,t) = \Delta A_s(E,t) + \Delta A_p(E,t). \tag{S8}$$

Supplementary Note 4: Global fitting procedure and results

We employ the following procedure to fit the experimental data.

1. At long time delays (>4 ps), we assume that the e_s electrons have equilibrated and the ΔA spectra at $t > 4$ ps are fit to Eq. S3 to yield the equilibrium transition energy $E_s(\infty)$ and the absorption coefficient $\epsilon_{\text{max},s}$, while fixing $W_{\text{G},s}$ and $W_{\text{L},s}$ to those reported in the literature.
2. Initial guesses of τ_{CB} and τ_p are obtained from fitting the ΔA time traces between 1.56 – 1.58 μm to an exponential rise and decay.
3. With the above initial guesses, we perform a global fit of the $\Delta A(E, t)$ data collected in the visible-NIR region to Eq. S5 to obtain $E_s(0)$, P_{dir}, and τ_{solv}.
4. Using the fit parameters obtained from the visible-NIR region, we employ Eq. S8 to fit the $\Delta A(E, t)$ data collected in the SWIR region. In this step, $E_s(\infty)$, $E_s(0)$, P_{dir}, and τ_{solv} are constrained to their respective values obtained from steps 1 and 3, while the parameters τ_{CB}, τ_p, $\epsilon_{\text{max},p}$, E_p, $W_{\text{G},p}$ and $W_{\text{L},p}$ are allowed to vary freely for the global fitting. Since the retrieved E_p is situated very close to the detector cut-off, we do not report $W_{\text{G},p}$, which characterizes the low-energy side of the p state absorption band.
5. Constraining τ_{CB} and τ_p to their values obtained from the global fitting in the SWIR, steps 3 and 4 are repeated until the values of the fit parameters converge.

The results of global fitting, obtained from 16 samples for H_2O and 64 samples for D_2O, are summarized in Supplementary Table 1.

Supplementary Table 1. Summary of results obtained from global fitting of time-resolved differential absorption spectra of ionized liquid H_2O and D_2O.

	H_2O	D_2O
$E_s(\infty)$ / eV	1.730 ± 0.019	1.760 ± 0.014
$E_s(0)$ / eV	1.047 ± 0.101	1.134 ± 0.040
E_p / eV	0.754 ± 0.031	0.752 ± 0.023
$W_{\mathrm{L},p}$ / eV	0.242 ± 0.014	0.252 ± 0.013
$\epsilon_{\mathrm{max},p}/\epsilon_{\mathrm{max},s}$	5.3 ± 1.6	2.7 ± 0.3
P_{dir}	$(2.22 \pm 0.01) \times 10^{-14}$	$(2.22 \pm 0.01) \times 10^{-14}$
τ_{CB} / ps	0.26 ± 0.02	0.26 ± 0.01
τ_p / fs	62 ± 10	110 ± 5
τ_{solv} / ps	0.37 ± 0.02	0.43 ± 0.03

Supplementary Note 5: SWIR excited-state absorption spectrum of the hydrated electron

We perform three-pulse experiments to record the excited-state absorption of the hydrated electron in the SWIR. In these experiments, the hydrated electron e_s is prepared by ionizing liquid water with an intense, visible-NIR laser pulse. After a time delay of 33 ps, permitting for equilibration of e_s, a weak, visible-NIR laser pulse is used to excite e_s to e_p, whose SWIR absorption signature is then recorded by a SWIR probe pulse. At a time delay of 40 fs, the ΔA spectrum slopes up towards the long-wavelength edge (Supplementary Figure 4). This spectral feature is similar to that observed in ionized liquid water (see Fig. 2b of the main text), thus supporting our assignment of the absorbing species responsible for Fig. 2b to the e_p electron.

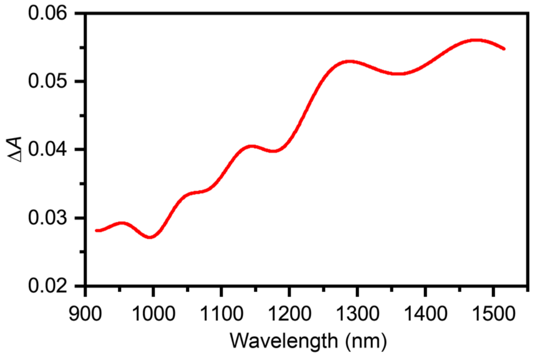

Supplementary Figure 4. SWIR absorption of the excited-state hydrated electron. SWIR differential absorption recorded 40 fs following the visible-NIR photoexcitation of the equilibrated hydrated electron, showing the increasing ΔA towards the long-wavelength region.

Supplementary Note 6: Laser spectra and interferometric autocorrelation

The spectra of the laser pulses employed in the experiment and the interferometric autocorrelation traces are shown in Supplementary Figures 5a – 5f. The pulse durations (FWHM) of the visible-NIR, visible, and SWIR pulses are 4.5 fs, 5.4 fs, and 8.5 fs, all corresponding to sub-two-cycle duration.

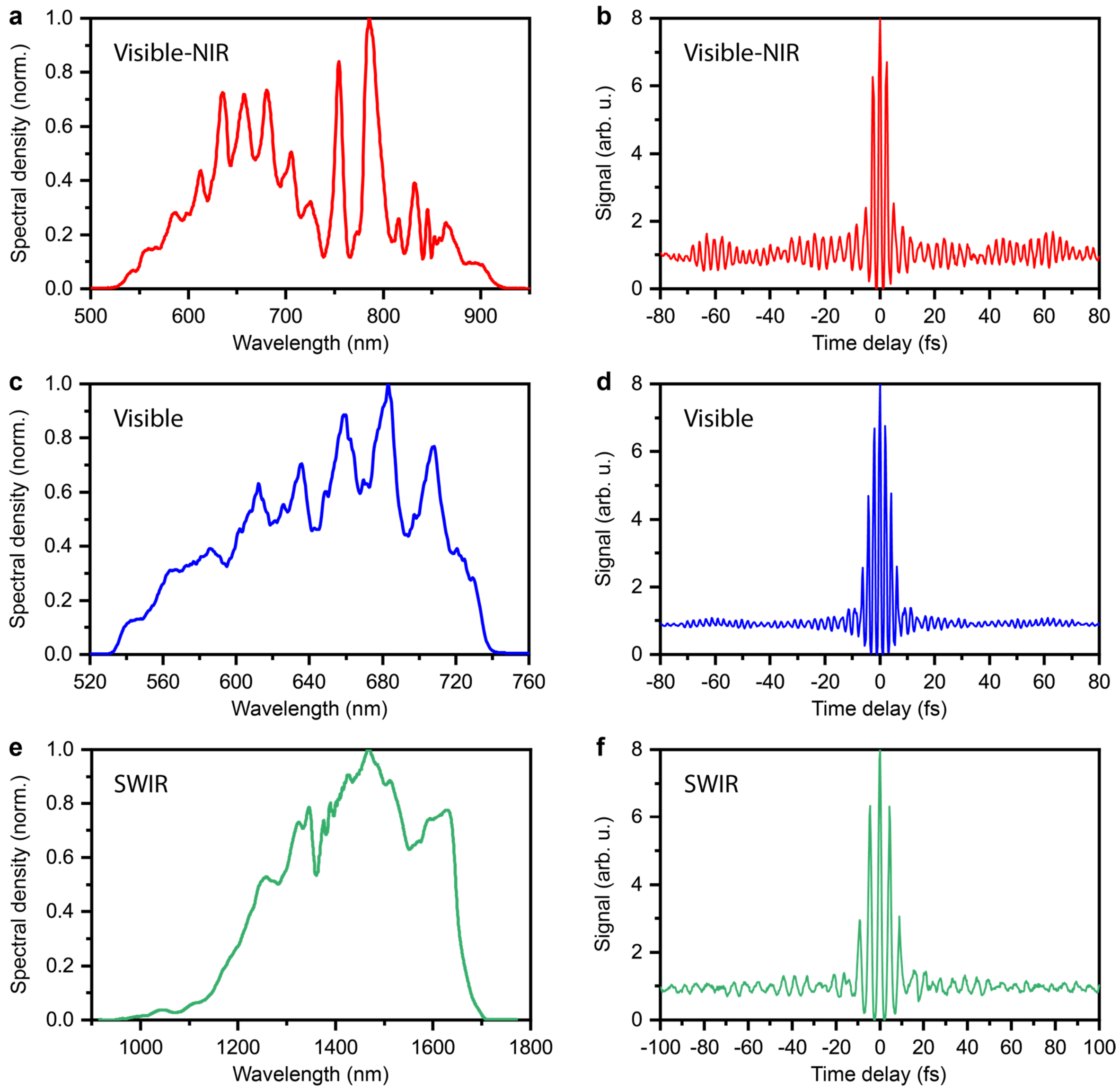

Supplementary Figure 5: Laser spectra and interferometric autocorrelation traces. (a) & (b) Visible-NIR pulse, **(c)** & **(d)** visible pulse, **(e)** & **(f)** SWIR pulse.

Supplementary Note 7: Long-pass filter of differential absorption spectra

Differential absorption spectra are processed by applying a Butterworth long-pass filter to remove spurious features caused by the presence of modulations in the probe spectra. The relatively wide absorption spectra of the hydrated electron justify this post-processing method. The gain function for the Butterworth filter is defined as

$$G(\nu^{-1}) = \frac{1}{\sqrt{1 + (\nu^{-1}/\nu_c^{-1})^{16}}}, \tag{S9}$$

where ν^{-1} is the inverse of the spectral frequency and ν_c^{-1} is the cut-off of the inverse spectral frequency. The long-pass filtered time-resolved transient absorption spectra are obtained by

$$\Delta A(\nu, t) = \mathcal{F}_\nu^{-1}\left(\mathcal{F}_\nu\big(\Delta A_{raw}(\nu, t)\big)G(\nu^{-1})\right), \tag{S10}$$

where $\mathcal{F}_\nu$ denotes Fourier transform with respect to the spectral frequency dimension and ΔA_{raw} is the raw time-resolved transient absorption spectra. The chosen cut-off inverse spectral frequencies are $\nu_c^{-1} = 20$ fs and $\nu_c^{-1} = 40$ fs for the data in the visible and SWIR regions respectively.

Supplementary References

1. Kerr G. D., Hamm R. N., Williams M. W., Birkhoff R. D., Painter L. R. Optical and dielectric properties of water in the vacuum ultraviolet. *Phys. Rev. A* **5**, 2523-2527 (1972).
2. Pépin C., Goulet T., Houde D., Jay-Gerin J. P. Observation of a continuous spectral shift in the solvation kinetics of electrons in neat liquid deuterated water. *J. Phys. Chem. A* **101**, 4351-4360 (1997).
3. Bin Mohd Yusof M. S., Lim Y. L., Loh Z.-H. Ultrafast vibrational wave packet dynamics of the aqueous tyrosyl radical anion induced by photodetachment. *Phys. Chem. Chem. Phys.* **23**, 18525-18534 (2021).
4. Bin Mohd Yusof M. S., Debnath T., Loh Z.-H. Observation of intra- and intermolecular vibrational coherences of the aqueous tryptophan radical induced by photodetachment. *J. Chem. Phys.* **155**, 134306 (2021).
5. Bin Mohd Yusof M. S., Siow J. X., Yang N., Chan W. X., Loh Z.-H. Spectroscopic observation and ultrafast coherent vibrational dynamics of the aqueous phenylalanine radical. *Phys. Chem. Chem. Phys.* **24**, 2800-2812 (2022).
6. Jou F.-Y., Freeman G. R. Temperature and isotope effects on the shape of the optical absorption spectrum of solvated electrons in water. *J. Phys. Chem.* **83**, 2383-2387 (1979).
7. Jortner J., Levine R., Ottolenghi M., Stein G. The photochemistry of the iodide ion in aqueous solution. *J. Phys. Chem.* **65**, 1232-1238 (1961).
